# Development and Characterization of a Stable Oil-in-Water Nanoemulsion Using Impingement Jet Mixing and Lyophilization Techniques

**DOI:** 10.3390/pharmaceutics18060745

**Published:** 2026-06-17

**Authors:** Anna Shao, Jingyan Zhang, Zhaowei Jin, Yao Li, Jialin Tang, Quanmin Chen, Hongbing Wu, Jeremy Guo

**Affiliations:** WuXi Biologics, Shanghai 200131, China; shao_anna@wuxibiologics.com (A.S.); zhang_jingyan@wuxibiologics.com (J.Z.); jin_zhaowei@wuxibiologics.com (Z.J.); li_yao3601@wuxibiologics.com (Y.L.); tang.jialin@wuxibiologics.com (J.T.); chen_quanmin@wuxibiologics.com (Q.C.); wu_hongbing@wuxibiologics.com (H.W.)

**Keywords:** nanoemulsion, impingement jet mixing, lyophilization, high shear homogenization, microfluidization

## Abstract

Nanoemulsion (NEM) is an effective adjuvant and delivery system for vaccines and nucleic acids, capable of inducing immune responses against diverse pathogens. **Background/Objectives**: Conventional NEM manufacture uses multi-step operations, typically high-shear homogenization and then microfluidization (HSHM), thereby increasing process complexity and contamination risk. As water-rich colloidal dispersions, NEM is prone to microbial proliferation and droplet coalescence; freezing further disrupts microstructure, causing phase fusion and separation, so NEM adjuvants are often stored separately from antigens in multi-vial formats. Lyophilization could reduce cold-chain dependence and enable single-vial products, but there is no systematic study on lyoprotectants comparation and process optimization of lyophilized NEM. **Methods**: An impingement jet mixing (IJM) process was evaluated as a simplified, scalable route for NEM production. Key IJM parameters, including flow ratio, total flow rate, preparation temperature, microchannel type, and shear mode—were examined to match attributes of conventional HSHM. Lyophilized and reconstituted NEM were characterized by dynamic light scattering, scanning electron microscopy, transmission electron microscopy, differential scanning calorimetry and/or in vitro potency to inform lyoprotectant selection, and Taguchi Design of Experiment (DOE) methodology guided lyophilization processes. **Results**: IJM yielded NEM with droplet size, polydispersity index (PDI) and morphology comparable to HSHM, with higher throughput and fewer unit operations. Optimized lyophilization technique with designed lyoprotectant and process formed closed structures to prevent the easy-to-flow monolayer of the emulsion from fusing, producing robust and stable NEM. **Conclusions**: Coupling IJM with targeted lyophilization establishes a scalable, lower-risk manufacturing paradigm for NEM that preserves critical quality attributes, reduces cold-chain reliance and enables single-vial adjuvanted vaccine formats with tangible industrial and clinical benefits.

## 1. Introduction

Emulsions are commonly classified by droplet size into macroemulsion (>500 nm) and nanoemulsion (NEM, 20–500 nm) [1]. NEMs are further described by their dispersed/continuous phase relationship as oil-in-water (O/W) or water-in-oil (W/O), with the continuous (greater volume) phase forming the external medium. When biphasic emulsions are dispersed in another oil or water phase, multiple NEMs, such as oil-in-water-in-oil (O/W/O) and water-in-oil-in-water (W/O/W), are formed.

Particularly, O/W NEM have been developed as vaccine adjuvants. They increase antigen retention at the injection site (depot effect), stimulate local production of pro-inflammatory cytokines and chemokines, induce damage-associated molecular patterns (DAMP)-signaling as well as recruit innate immune cells such as neutrophils, eosinophils, dendritic cells, monocytes, and macrophages. NEM are commonly mixed with a preformed antigen prior to administration; the NEM adjuvant and the antigen are stored independently and combined immediately before dosing. Even without physical encapsulation, spatial co-localization of NEM adjuvant and antigen at the injection site can enhance antigen presentation and immune activation by promoting recruitment, uptake, and migration of antigen-presenting cells.

**Figure 1:**
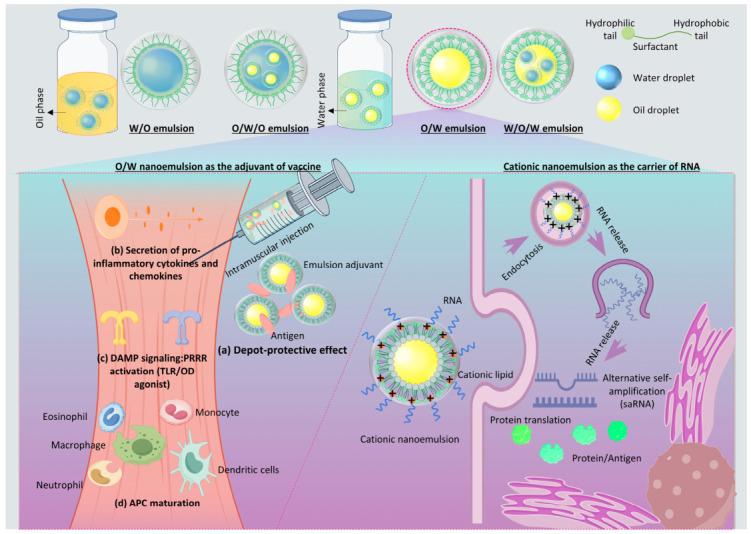
Schematic illustration of different NEMs and common applications as adjuvant and RNA carrier of O/W NEM.

Examples of NEM adjuvants include MF59, AS03, and alum-stabilized Pickering emulsion (PAPE) [2]. Cationic nanoemulsion (CNEM), by electrostatic complexation with nucleic acids can enhance cellular uptake, translation efficacy, and immune response [3] (Figure 1). MF59 is an O/W emulsion composed of squalene, polyoxyethylene sorbitan monooleate (Tween 80), and sorbitan trioleate (Span 85), with a mean particle size of about 165 nm [4,5]. MF59 has been approved for use in human influenza vaccines in more than 30 countries [6,7]. CNEM is an MF59-based O/W formulation modified by inclusion of a cationic lipid that adsorbs RNA onto the oil-droplet surface through electrostatic interactions with the negatively charged phosphate backbone. The cationic nanoemulsion-delivered self-amplifying RNA (saRNA-CNEM), with its nanoscale size (100–250 nm range) and positive charge, can elicit anticipated immune responses against various infectious targets such as influenza virus, HIV, cytomegalovirus, and malaria in pre-clinical animal studies [8,9,10]. CNEM, as an RNA delivery vector, has the advantage of promoting local innate immune activation and immune-cell recruitment, similar to an MF59 adjuvanted subunit vaccine [9], and can also be prepared and stockpiled separately from the RNA for later use.

The NEM adjuvant with a particle size around 80–250 nm has a strong ability to induce both cellular and humoral immunity. Empirical comparisons have shown a size-dependent effect on immunogenicity; specifically, a nano-sized emulsion with an average diameter of 214 ± 54 nm generated higher antibody titers than a micron-sized emulsion with the same components and an average diameter of 1080 ± 360 nm [11,12,13,14].

**Figure 2:**
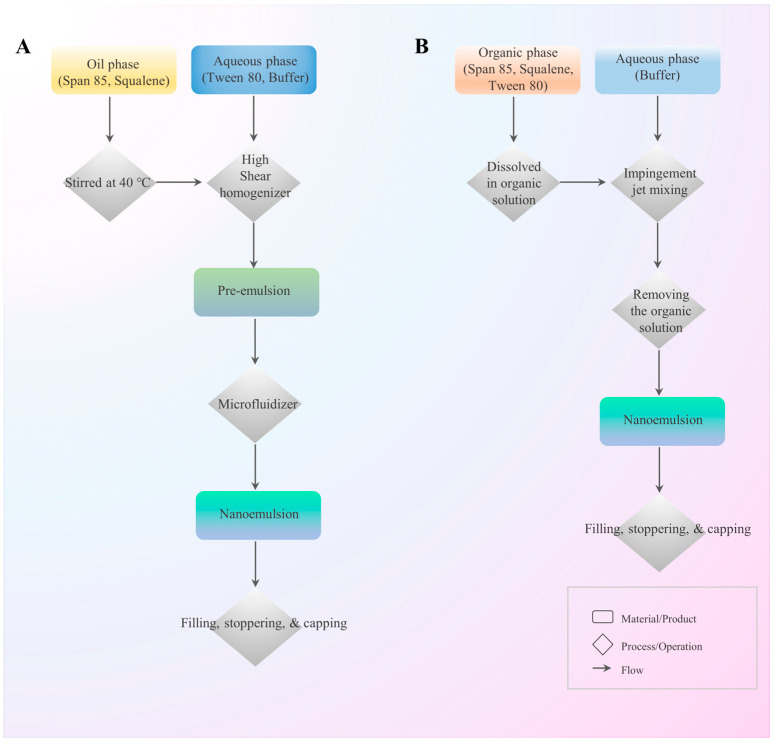
Outline of O/W NEM manufacturing process by (**A**) HSHM and (**B**) IJM.

High shear homogenization and then microfluidization (HSHM), as shown in Figure 2, is a widely used two-step approach to produce the long-term stable MF59-based NEM [15]. In the first step, intense shear from instruments generates a primary O/W emulsion. The primary emulsion is then forced through microfluidizer channels at high pressure to create high-velocity liquid streams. However, HSHM typically involves multiple stainless-steel vessels and transfer lines for heating, mixing, and pressurization, increasing product contact surfaces and opportunities for environmental exposure. According to the GMP (Good Manufacturing Practice) regulations, all product-contact equipment requires validated cleaning, maintenance, and sanitation to prevent contamination. At the same time, open or complex processing will introduce RNases—enzymes that are widespread in air and highly active against RNA, leading to the instability of saRNA-CNEM formulations and limiting the application of NEM as nucleic acid carriers.

Impingement jet mixing (IJM) generates two high-velocity fluid streams that collide, producing intense mixing, shear, and rapid homogenization [16]. It has been widely used for mRNA delivered by lipid nanoparticles (mRNA-LNPs) production. When implemented as a closed single-use system, IJM enables aseptic, low-RNase processing with minimal cleaning/validation burden. Moreover, the widespread application of this process—which allows for parallel scaling—enables the production of uniform and stable products, thereby further facilitating industrialization. Farahnaz et al. prepared an emulsion with particle size below 100 nm by dissolving hemp seed oil and surfactants of Tween 80 and sorbitan monooleate (Span 80) in ethanol as the organic phase and impinging it against deionized water through opposing channels, which is based on the similar principle to IJM [17], and supports the applicability of IJM for NEM formation.

Biologics require low-temperature storage to preserve potency, but freezing and excessive heat both threaten NEM integrity and antigen stability [18]. The high water content of NEM raises concerns about microbial growth, encapsulated aggregation, and leakage, thereby reducing therapeutic efficacy and shortening the shelf life [19]. Lyophilization removes water from frozen samples by sublimation, converting an aqueous solution or suspension into a more chemically and physically stable solid. For NEM-based vaccines, lyophilization can allow co-formulation of antigen or RNA with the emulsion prior to storage, reducing separate manufacturing and cold-chain costs [20]. Nevertheless, lyophilization imposes distinct stresses due to the crystallization of the oil and aqueous phases. Ice formation can concentrate solutes and alter local ionic strength and pH, and interfacial dehydration can disrupt surfactant films, promoting droplet coalescence and aggregation, as well as compromising the integrity of NEM [21].

Although lyophilized vaccines account for one-quarter of FDA-approved vaccines, no licensed single-vial lyophilized vaccines containing adjuvants are currently available [22], partially because of challenges in co-stabilizing adjuvants with antigens during lyophilization. Thin-film freeze-drying (TFFD) technology can produce single-vial, freeze-insensitive dry powders [23,24]. But TFFD requires specialized equipment and technology compared to conventional shelf lyophilization, resulting in higher equipment and operation costs, greater process complexity, and additional scale-up and manufacturing challenges. Iyer et al. demonstrated that conventional shelf lyophilization of the Medimmune emulsion produced only an approximate 20 nm increase in particle size after reconstitution [25]. Preston KB et al. showed that co-lyophilizing a squalene-in-water emulsion, with the formulation containing a high concentration of trehalose, could withstand temperatures up to 40 °C for 12 weeks [26]. Orr developed a single-vial tuberculosis vaccine containing antigens and NEM that remained stable at sustained elevated temperatures [20]. Based on these studies, conventional shelf lyophilization can yield stable lyophilized NEM products, provided the lyophilization process and formulation are carefully optimized to preserve colloidal structure and biological activity.

Our study provides the first systematic investigation to design a novel IJM process that produces NEMs comparable to those from the HSHM process. We optimize IJM operating variables, including total flow rate, flow rate ratio, microchannel type, shear mode, and mixing temperature, and evaluate formulation and lyophilization conditions for MF59-based and cationic MF59-derived NEMs. Lyophilization variables include choice of protectants, freezing ramp rate, primary-drying temperature, and ramp rate. Product attributes used to define optimal process and formulation are Z-average particle size, polydispersity index (PDI), transmission electron microscopy (TEM), squalene content, scanning electron microscopy (SEM), lyophilized cake shape, reconstitution time, residual moisture, and in-vitro potency. These metrics guide selection of IJM settings and lyophilization parameters, and enabled comparative stability assessment of liquid and lyophilized NEMs prepared by different processes.

## 2. Materials and Methods

### 2.1. Materials

All chemicals were used as received without any further purification. Sodium dihydrogen phosphate dihydrate, disodium hydrogen phosphate dihydrate, isopropanol, and sorbitol were obtained from Merck (Darmstadt, Germany). Tween 80 was purchased from J.T. Baker (Radnor, PA, USA). Squalene and Span 85 were acquired from Sigma–Aldrich (Darmstadt, Germany). Sucrose, mannitol, and trehalose were supplied by Pfanstiehl, Inc. (Waukegan, IL, USA).

### 2.2. Preparation of Liquid NEM or CNEM

#### 2.2.1. High Shear Homogenization and Microfluidization (HSHM)

For the classical HSHM process, the aqueous phase was prepared by dissolving 0.47 g Tween 80 in 94.52 g sodium citrate buffer (10 mM, pH 6.5) with stirring at 40 °C. The oil phase was prepared by dissolving 0.47 g Span 85 into 3.90 g squalene under stirring at 40 °C, then the pre-emulsion was generated by mixing the aqueous and oil phase at 8000 rpm for three cycles, using high shear homogenizer IKA T25 Ultra-Turrax (IKA, Wilmington, NC, USA). The final NEM was obtained by homogenizing the pre-emulsion at 750 bar for 10 cycles using the microfluidizer, Nathox Lab 3 (NTI, Pittsburgh, PA, USA).

With the composition and process listed above, the final CNEM was obtained by adding 0.4 g DOTAP (1,2-dioleoyl-3-trimethylammonium-propane) in the oil phase.

#### 2.2.2. Impingement Jet Mixing (IJM) Process

An IJM device based on T-junction was constructed for the fabrication of NEM. It was composed of two syringe pumps (Pump 11 Elite, Harvard Apparatus, Inc., Holliston, MA, USA), three pieces of PEEK tubing (inner diameter 1.0 mm), a microchannel made of PEEK (inner diameter 0.5 mm), and an emulsion collector. The syringe pumps were used for the delivery of the aqueous phase and organic phase through the PEEK tubing, respectively, and all syringe pumps were equipped with a 50 mL plastic syringe. Next, 3.90 mass of squalene, 0.47 mass of Tween 80, and 0.47 mass of Span 85 were dissolved in IPA to prepare the organic phase solution, which was then mixed with the 94.52 mass of sodium citrate buffer (10 mM, pH 6.5) through the microchannel, in which the effects of the flow rate ratio (1~4:1), flow rate (63.1, 85.8, 122.4 and 137.1 mL/min), microchannel type (T-shaped, arrow-shaped, Y-shaped), the mixing temperature (25 °C, 40 °C and 60 °C) and the shear mode on the emulsion were investigated. Finally, IPA was removed from the system by dialysis to obtain the final NEM.

### 2.3. Preparation of Lyophilized NEM

NEMs were mixed with the designed agent solutions in sodium citrate buffer (10 mM sodium citrate, pH 6.5) at the same volume ratio. A 0.5 mL aliquot of the liquid solution was filled into a 2 mL vial, half stoppered, and then loaded into the lyophilizer. The liquid formulation was converted to dry powder using conventional shelf lyophilization with a Lyostar III (SP Scientific, Warminster, PA, USA) under different conditions, with parameters listed in Table 1.

Briefly, during the early feasibility study, an initial lyophilization cycle for NEMs with or without 10% sucrose was implemented based on prior knowledge and experience with common products. The cycle was conservatively designed well below the collapse temperature (Tc). Optimization of lyophilization parameters for NEM with 10% sucrose and 1% mannitol was achieved using Taguchi experimental design with three different factors (freezing ramp time, primary drying temperature, primary drying ramp time), each at three different levels, arranged in an orthogonal layout of L9 as shown in Table 1 by Minitab 21. At the end, one set of effective parameters was applied in the studies of lyoprotectant agents, NEMs and CNEMs stability with different lyoprotectant compositions listed in Table 2.

### 2.4. Stability Study of Liquid and Lyophilized NEM

To investigate the stability of the liquid NEMs prepared by IJM and HSHM, freshly prepared liquid emulsion samples were stored at 4 °C and 25 °C, and the appearance, Z-average, PDI, and the content of squalene were examined at T0, week 1, week 2, and month 1.

In order to compare the stability of the lyophilized NEMs prepared by IJM and HSHM, NEMs were added with either 10% sucrose or 10% sucrose + 1% mannitol, and then lyophilized according to the stability study procedure in Table 1. The lyophilized NEMs were stored at 4 °C and 25 °C. The cake shape, residual moisture, reconstitution time, Z-average, PDI, and the content of squalene were examined at week 1, week 2, and month 1, respectively.

Furthermore, to compare the stability of liquid versus lyophilized NEMs, lyoprotectants listed in Table 2 were added to the NEM and CNEM samples and lyophilized following the stability study procedure in Table 1. Both liquid and lyophilized NEMs and CNEMs were stored at 5 °C, 25 °C, and 40 °C at week 1, week 2, and month 1 for short-term and accelerated stability study. For long-term storage stability, we selected the liquid and lyophilized NEMs at 5 °C and 25 °C for month 3 evaluation and at 5 °C for month 32 evaluation. All the samples were tested by Z-average and PDI.

### 2.5. Characterization

#### 2.5.1. Freeze Drying Microscopy of NEM

The freeze-drying microscopy (Lyostat5, BioPharma, Winchester, UK) of the lyophilized NEM was examined by fast cooling to −60 °C at the rate of 10 °C/min. Then, the vacuum pump was turned on, and the temperature was increased to −45 °C at the rate of 5 °C/min. Then, the heating rate was adjusted to 2 °C/min until a significant collapse appeared in the lyophilized sample. Images were captured every 2 s in the whole process.

#### 2.5.2. Scanning Electron Microscopy (SEM) of Lyophilized NEM Cake

The morphology of lyophilized NEM was examined using a field emission SEM instrument (SU8010, Hitachi, Tokyo, Japan) at an accelerating voltage of 3.0 kV and magnifications ranging from 50× to 450×. The sample pieces were cut from the lyophilized cake center without structural destruction and were sputter-coated with gold (Au) to achieve a 5–10 nm Au layer thickness.

#### 2.5.3. Residual Moisture Assay of Lyophilized NEM Cake

The sample was fast crushed and transferred into the anolyte reservoir immediately, then the moisture content was determined automatically by Karl Fischer Titrator (C30S, Mettler Toledo, Columbus, OH, USA) at 15–30 °C and less than 50% environmental humidity.

#### 2.5.4. Reconstitution of Lyophilized NEM Cake

The NEM after lyophilization was reconstituted by adding 0.5 mL of deionized water with gently agitation, then the rehydrated aqueous emulsion was further characterized.

#### 2.5.5. Analysis of Z-Average Particle Size and Polydispersity Index (PDI) of NEM and Rehydrated Aqueous Emulsion

Z-average and PDI can be measured by both Malvern particle size analyzer (ZSU3200, Malvern, Worcestershire, UK) and Wyatt dynamic light scattering plate reader (Dynapro^TM^ Plate Reader III, WYATT TECHNOLOGY, Santa Barbara, CA, USA). Different instruments have varying data outputs with the specific analyzing methods/reference samples, even for the same emulsion dilution. Z-average data measured by Malvern are slightly larger (~20 nm) than that of Wyatt.

The NEM samples were diluted 10 to 100 times with deionized water prior to conducting the particle size and PDI measurement. During the NEM preparation process study, the Z-average and PDI were measured by Malvern. For the other studies, samples were measured and compared with Z-average and PDI by Wyatt.

#### 2.5.6. Transmission Electron Microscopy (TEM) Measurements of NEM and Rehydrated Aqueous Emulsion

The microscopic morphology of the NEM was examined with a transmission electron microscopy (JEM-1400flash, JEOL, Tokyo, Japan). Samples were placed on a copper grid, stained with 1% uranyl acetate, and air dried at room temperature, then observed at 80 kV.

#### 2.5.7. Squalene Content of NEM and Rehydrated Aqueous Emulsion

Referring to the method of Sundaramurthi et al. [27], the concentrations of squalene were analyzed by HPLC-ELSD. The equipment used was the Agilent HPLC (Agilent Technologies, Santa Clara, CA, USA) 1260 Infinity II system with an AdvanceBio Peptide Map C18 column (2.7 μm, 120 A, 2.1 mm × 150 mm) and an evaporative light scattering detector (ELSD). Mobile phase A and B were 50 mM ammonium acetate at pH 4 and acetonitrile, respectively, at a flow rate of 0.5 mL/min, and column temperature was maintained at 28 °C. The elution gradient was initiated at 90% B and maintained for 1 min, and then increased to 97% B in 19 min, and maintained at 97% B for 25 min. Finally, the column was equilibrated at 90% B for 5 min. The ELSD parameters were set with evaporator temperature of 60 °C, nebulizer temperature of 90 °C, and gas flow rate of 1.6 SLM.

A squalene stock solution of 5 mg/mL in 100% IPA was prepared. Serial dilutions were made from the stock solution to the concentrations of 1 mg/mL, 0.8 mg/mL, 0.4 mg/mL, 0.2 mg/mL, and 0.1 mg/mL. Test samples were diluted 100-fold in 80% IPA. Squalene standard solutions and diluted samples were injected in 10 μL volumes.

#### 2.5.8. Differential Scanning Calorimetry (DSC) of Lyophilized NEM

The heat flow measurements were performed by DSC (DSC 2500, TA Discovery, New Castle, DE, USA). The samples, both the excipient powder and the cake after lyophilization, were placed in 40 μm aluminum pans and sealed, then were subjected to a program with equilibration at 25 °C for 5 min, heating 10 °C/min to 200 °C. A sealed empty pan was used as the reference.

#### 2.5.9. In Vitro Potency Assessment of NEM Containing Ovalbumin (OVA)

OVA stock solutions containing different lyoprotectants were mixed with MF59 at a 1:1 (*v*/*v*) ratio to produce OVA-loaded NEMs with a final OVA concentration of 0.25 mg/mL (the selection of lyoprotectants is shown in Table 2). A 0.5 mL aliquot of the liquid solution was filled into a 2 mL vial, half stoppered, and then placed in the lyophilizer.

After lyophilization and reconstitution, samples were diluted with sample diluent. All reagents were warmed to room temperature before use and mixed gently to avoid foaming. First, 100 μL horseradish peroxidase (HRP)-conjugate was dispensed into each well of a microtitration plate that had been coated with polyclonal anti-ovalbumin antibodies. Then, 100 μL of diluted samples, standards, and control were added to the intended wells and mixed gently. Standards and control were run in duplicate, and diluted samples were run in triplicate. The plate was covered and incubated for 60 min at 37 °C. The wells were then emptied, washed five times with 300 μL wash solution, and the plate was tapped dry on absorbent paper. Next, 100 μL of 3,3′,5,5′-tetramethylbenzidine (TMB) substrate was dispensed into each well and incubated for 10 min at room temperature, protected from light. Finally, 100 μL of stop solution was dispensed into each well and mixed gently. The optical density at 450 nm was read with a microplate reader within 30 min after reaction stop.

## 3. Results and Discussion

### 3.1. IJM Process Parameter Design

In IJM process, flow rate ratio, total flow rate, microchannel type, temperature, and shearing mode are the key parameters affecting the particle size and PDI of NEM. In general, changes in flow rate ratio influence the droplet size by affecting the shear effect between the two phases and the interfacial tension. The total flow rate, on the other hand, regulates particle size by affecting the fluid pattern, mixing efficiency, and coalescence within the channel [28]. In addition, various preparation temperatures further affect the particle size and morphology by changing the viscosity of the system or the solubility of the components [29,30]. The breakup process generated by various shear modes within the microfluidic devices also impacts the droplet size and polydispersity [31]. Therefore, the above four key parameters were investigated in this study.

**Figure 3:**
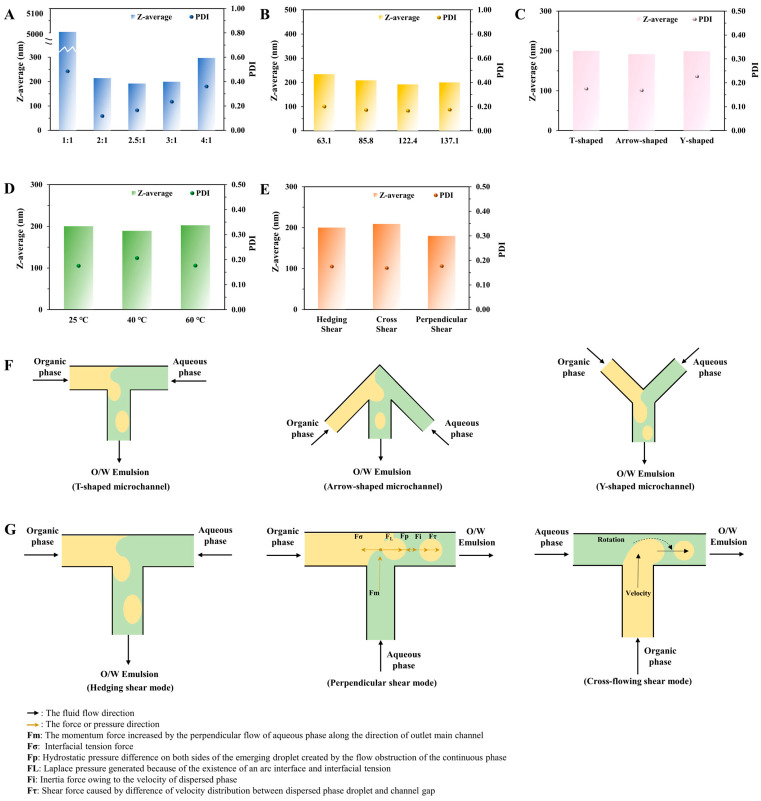
IJM process parameter investigation of NEM. (**A**) Flow rate ratio, (**B**) Total flow rate, (**C**) Microchannel type, (**D**) Preparation temperature, (**E**) Shear mode, (**F**) Schematic diagrams of various microchannel types, and (**G**) Schematic diagrams of various shear modes.

First, the effect of different flow rate ratios of aqueous and organic phases in preparation on emulsion droplet size and PDI was studied, as shown in Figure 3A. When the aqueous-to-organic phase flow rate ratio was increased from 1:1 to 2.5:1, the Z-average size of the emulsion gradually decreased below 200 nm, and the PDI also dropped below 0.3. On the contrary, the emulsion Z-average and PDI tended to increase when the flow rate ratio further increased from 2.5:1 to 4:1. It is considered that the increase in the flow rate ratio within an appropriate range leads to a decrease in droplet size, due to both a faster process of mixing and an increased dilution effect that consequently accelerates the diffusion between the aqueous and organic phases [17]. However, if the aqueous phase flow rate is increased excessively, insufficient shear and mixing between the two phases may occur due to the large difference in the flow rate, therefore, a NEM with great particle size performance could not be formed [32]. Overall, 2.5:1 was the optimal flow rate ratio in the microfluidic process and it would be used in further study.

Total flow rate was the next process parameter investigated. As shown in Figure 3B, Z-average size and PDI were both decreased as the total flow rate increased. Higher flow rate, and correspondingly higher shear energy, facilitates the formation of smaller particles [33]. The Z-average size of the emulsion prepared was 200.2 nm when the total flow rate was controlled at 137.1 mL/min.

The particle size and PDI of NEM are also influenced by the type of microchannel. By tuning the microchannel geometry, the flow behavior can be altered, thereby affecting the contact, shear, and mixing efficiency between the two phases. Therefore, three types of microchannels, T-shaped, arrow-shaped, and Y-shaped, were designed in this study (Figure 3C,F). According to the data, all three microchannel types could produce emulsions with small particle sizes, but the Y-shaped exhibited a relatively higher PDI. The convergence angle of the microchannel (i.e., the deviation angle of the two-phase inlets relative to the emulsion outlet direction) affects droplet breakup mechanisms, and thus determines the emulsion droplet size distribution. In the arrow-shaped geometry, which has the largest convergence angle (>90°), a locally stable and concentrated high-shear region is generated, causing the liquid thread to break at a relatively fixed position. Its breakup mode is relatively uniform and highly repeatable. Although the T-type channel has an orthogonal convergence, the resulting squeezing effect and lateral shear also confine the breakup location, so it likewise produces a narrow size distribution. In contrast, the Y-type has the smallest convergence angle, so the oil and water phases retain substantial lateral momentum. Its breakup location and mode are more prone to fluctuation (for example, being squeezed off, dripping, or stretching into a fine jet before breaking), which leads to a higher PDI. In addition, emulsion formation in arrow-shaped microchannels was observed to require higher driving pressures. This is likely because the two phases encounter more concentrated high shear at the junction, leading to larger pressure drops compared with T- or Y-shaped channels. Consequently, for the same inner diameter, a higher-pressure-capacity infusion pump is needed for arrow-shaped microchannels. Therefore, after comprehensive consideration, the T-shaped was chosen as the most suitable microchannel type [34]. The preparation temperature also affects the physicochemical properties of the emulsion prepared by microfluidic technology (Figure 3D). When the temperature increased from 25 °C to 40 °C, the emulsion Z-average size decreased by about 11 nm. This may be because the viscosity of the organic phase decreases at higher temperatures, promoting the formation of smaller droplets. In contrast, when the temperature was further increased to 60 °C, the emulsion Z-average size increased to 202.5 nm. Based on the results obtained, a flow rate ratio of 2.5:1, a total flow rate of 137.1 mL/min, and a temperature of 40 °C were identified as the optimal parameters for emulsion preparation.

In addition to the flow rate ratio, total flow rate, microchannel type, and temperature, different fracture kinetics generated by various shear modes cause the neck of the organic phase breakup to form droplets, thereby affecting the size and polydispersity of the emulsion. Besides the hedging shear mode used previously, cross-flowing shear and perpendicular shear modes were also investigated (Figure 3E,G). In perpendicular shear mode, the organic phase was perpendicularly sheared by the aqueous phase when the aqueous phase was injected vertically and the organic phase was injected horizontally in a T-junction microchannel. As for the cross-flowing shear mode, the aqueous phase was injected in the horizontal direction, the organic phase was injected in the vertical direction, and the aqueous phase sheared the organic phase horizontally. The results showed that the minimum particle size was obtained by perpendicular shear mode at a total flow rate of 137.1 mL/min. The emulsion droplet had a homogeneous size distribution as the PDI was less than 0.2. The reason could be that in cross-flowing shear mode, the main forces causing the neck-break of the droplet were pressure drop, shear force, and inertial force, whereas in perpendicular shear mode, the momentum force, also called perpendicular shearing force, played an important role [31]. The organic phase in microchannels is known to undergo frequent rotational behavior after dispersing into small droplets under high-speed shear; however, rotation is an unstable state that easily leads to refusion and aggregation between adjacent droplets. Compared with cross-flowing shear, the momentum force in the perpendicular shear mode could reduce the rotational behavior of the droplet, thus facilitating the improvement of droplet stability [35].

### 3.2. Comparative Characterization and Stability of NEMs Prepared by IJM and HSHM

**Figure 4:**
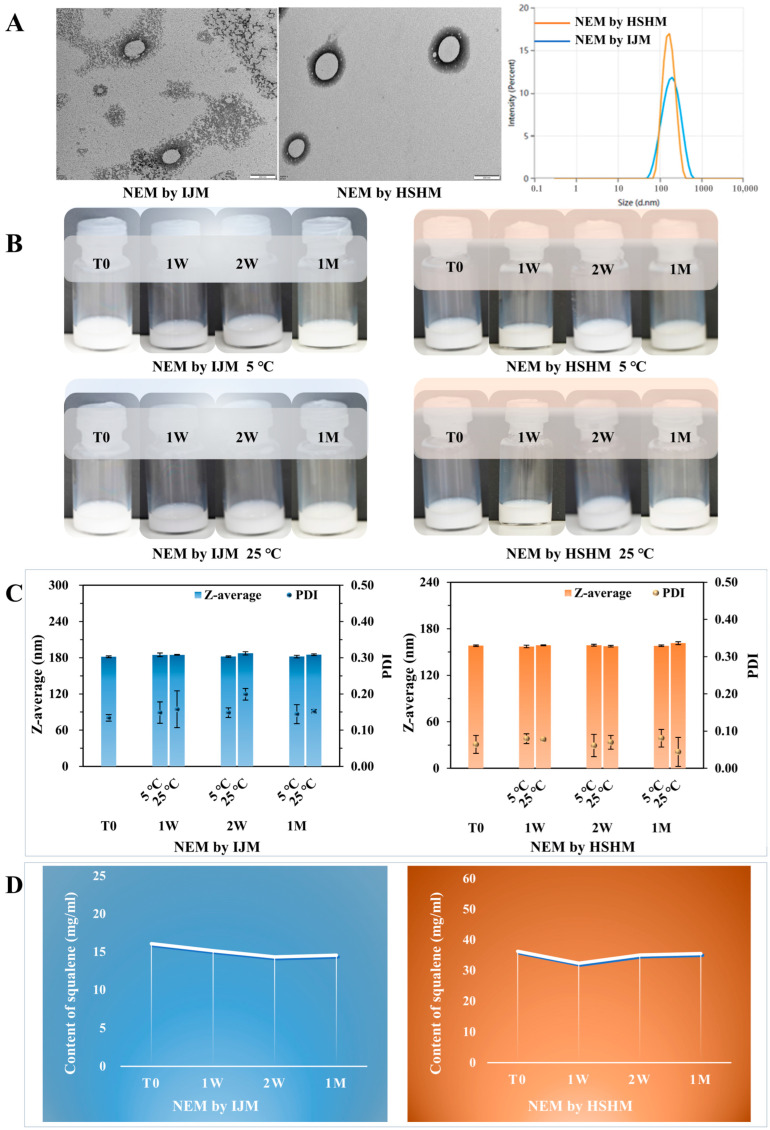
Comparison of NEMs by IJM and HSHM prepared by high-pressure homogenization and microfluidic process. (**A**) TEM images and size distribution of emulsions, (**B**) Appearances, (**C**) Z-average particle size and PDI, and (**D**) Squalene content change of emulsion in short-term stability study.

NEM was prepared using the optimized microfluidic process parameters described above, and the target product NEM by IJM was obtained after removing IPA by dialysis. There was no significant difference in TEM images and size distribution between NEMs prepared by IJM and HSHM (Figure 4A).

To further compare the emulsions produced by these two processes, the stability of emulsions was also studied. After 1 month of storage at 5 °C and 25 °C, both NEMs by IJM and HSHM retained a homogeneous milky appearance without delamination (Figure 4B). These results indicated that both processes were able to provide sufficient shear force to disperse the oil phase into uniform emulsion droplets during emulsion preparation. The same conclusion would be drawn by analyzing the particle distribution changes of the two emulsions during storage. Z-average size and PDI did not change for NEMs by IJM and HSHM when stored at different conditions for 1 week, 2 weeks, and 1 month (Figure 4C). Z-average size remained at 150–190 nm for both emulsions and PDIs were less than 0.3, indicating a homogeneous distribution. Squalene, as a commonly used oil component in O/W emulsions, is a straight-chain unsaturated triterpenoid with strong biological activity. Emulsions of squalene combined with surfactants can form efficient adjuvants when added to vaccines [2]. Obviously, the change of squalene content of emulsions during storage is also one of the important indicators for stability. As adjuvants are usually stored and transported at 2–8 °C, neither NEM by IJM nor HSHM showed significant changes in squalene content after one month at 5 °C in this study (Figure 4D). Considering appearance, particle size, distribution, and squalene content, the NEMs prepared by both processes were considered stable and consistent.

The parameters investigated for IJM devices provide an alternative method for NEM preparation. It would be worth adding that parallelization or numbering-up—i.e., assembling multiple mixers with identical geometry and operating parameters in parallel as continuous production units to increase throughput—has become one of the mainstream strategies for scaling up IJM-mediated nanomedicine delivery systems. Parallel scale-up has inherent advantages in preserving the local shear field of single channels, thereby reducing nonlinear process effects associated with equipment scale-up. That also makes the process more valuable for commercial NEM preparation.

### 3.3. Investigation of Freeze-Drying Protectants

Emulsion droplet size is critical to maximize the effectiveness of the adjuvant and activate the correct immune response for vaccine efficacy [36]. Maintaining droplet size after lyophilization and reconstitution ensures maximum adjuvant effect [37]. When the percent polydispersity is greater than 30%, the particle population is considered polydisperse, containing significantly different sizes. Thus, in the multimodal distribution with a PDI over 0.3, the Z-average is highly sensitive to high intensity scatterers and the results of Z-average carry relevant size information. Therefore, it is vital to ensure that the droplets of NEM remain uniform and controllable in size after lyophilization and subsequent reconstitution.

**Figure 5:**
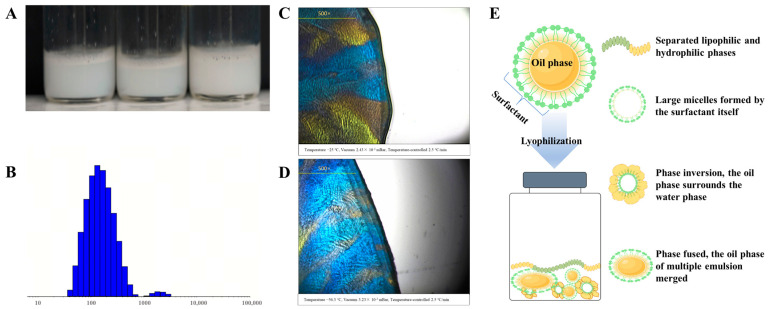
The characteristics of lyophilized and rehydrated blank emulsions without any excipients. (**A**) Appearance of rehydrated aqueous emulsion, (**B**) Particle size distribution graphic of rehydrated aqueous emulsion, (**C**) Freeze-drying microscopy images at T = −25 °C and (**D**) T = −56.3 °C, (**E**) The potential impact of lyophilization on the structure of emulsions.

At the beginning, blank NEM without any excipients was lyophilized to observe its changes in the process. Due to the high oil content, blank NEM was difficult to freeze even at temperatures below −55 °C, and no collapse status was captured between −56 °C and −25 °C (Figure 5C,D). After lyophilization, blank NEM did not form a cake and reconstituted into a solution that appeared turbid, with some insoluble particles visible (Figure 5A), and PDI showed double peaks with an aggregation peak larger than 1000 nm (Figure 5B). This proved that the structure of emulsion droplets was damaged during lyophilization. Hanson also pointed out that freezing NEM can lead to phase separation between lipophilic and hydrophilic phases, and thus adversely affect its structure [37]. Moreover, large micelles formed by the surfactant itself, phase inversion where the oil phase surrounds the water phase, or phase fused with the oil phase of multiple emulsion merged, resulting in a multimodal size distribution and structure changes (Figure 5E).

Lyoprotectants can change the particle–excipient interactions during the freeze-drying process. In the presence of sugar excipients such as sucrose or trehalose, they stabilize the formulation and help to achieve isotonicity after rehydration during the freeze-drying process [38]. Freeze-drying lyophilization has three main steps: freezing, primary drying, and secondary drying. The freezing phase is known to generate stresses, which has an impact on particle aggregation [39]. The primary drying phase is to remove ice by sublimation, which is the key step to forming different porous structures and has an impact on particle size after reconstitution [40]. Therefore, the temperature and sublimation rate in this phase should be well adjusted.

**Figure 6:**
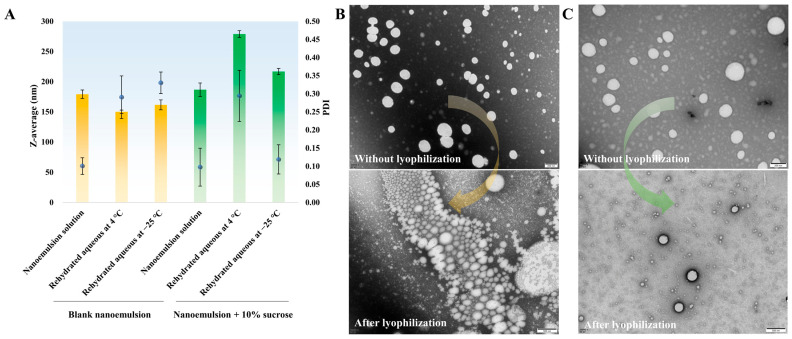
Preliminary study results included (**A**) Z-average particle size and PDI of the blank NEM and NEM with the content of 10% sucrose without lyophilization and after lyophilization with primary drying temperatures of −25 °C and 4 °C. TEM images of the (**B**) blank NEM and (**C**) NEM with the content of 10% sucrose without lyophilization and after lyophilization.

From the preliminary study, both blank NEM and NEM with 10% sucrose lyoprotectant dilution were lyophilized with the primary drying temperature at 4 °C and −25 °C. As shown in Figure 6A, after lyophilization without lyoprotectants, the particle size of reconstituted dilution was multimodally distributed with PDI up to 0.3, both at the primary drying temperature of 4 °C and at −25 °C. However, with the addition of 10% sucrose, multimodal distribution observed at primary drying temperature of 4 °C changed to monomodal distribution at −25 °C. Therefore, lyoprotectants and an optimized lyophilized process are necessary, as they coat emulsion droplets and protect NEM adjuvants from aggregation induced by freezing. The similar phenomena were also observed in TEM morphology. We found that in the lyophilized emulsion with the addition of sucrose (Figure 6C), almost all particles were evenly distributed in the field of view and retained the same size as before lyophilization. In contrast, the blank NEM became larger and severely aggregated after lyophilization (Figure 6B), which may be due to the membrane fusion and separation. All of these results directly indicate the positive influence of lyoprotectants.

AboulFotouh et al. [41] designed various concentrations of sucrose, trehalose and mannitol as lyoprotectants for thin-film lyophilization of vaccines containing NEM. As a result, sucrose and trehalose performed better than mannitol. Sucrose and trehalose are commonly used as lyoprotectants in the pharmaceutical industry. The interaction between hydroxyl groups in their structures and water molecules increases the viscosity of the solution and displaces water molecules during the drying process. It will avoid aggregation of the surfactant layer on the surface of droplets, thereby reducing mechanical stress and formation of crystalline [42]. In addition, Gao et al. [43] demonstrated that sucrose, trehalose, and lactose as lyoprotectants could stabilize nanoliposomes containing squalene in lyophilization. Crystallization of mannitol during freezing may cause destruction of the emulsion during sublimation. It is possible that the incorporation of mannitol at a low content (typically ≤1% *w*/*v*) in formulations increases the cake mass and prevents blowout during the lyophilized process. Under the hard conditions of lyophilization, lyoprotectants at various concentrations may have different effects on samples, with higher concentrations generally performing better [44]. Based on that, the lyoprotectant agents study was designed using various concentrations of sucrose, trehalose, mannitol, sorbitol, and two-component lyoprotectant mixtures as protectants for lyophilization of NEM in this study.

**Figure 7:**
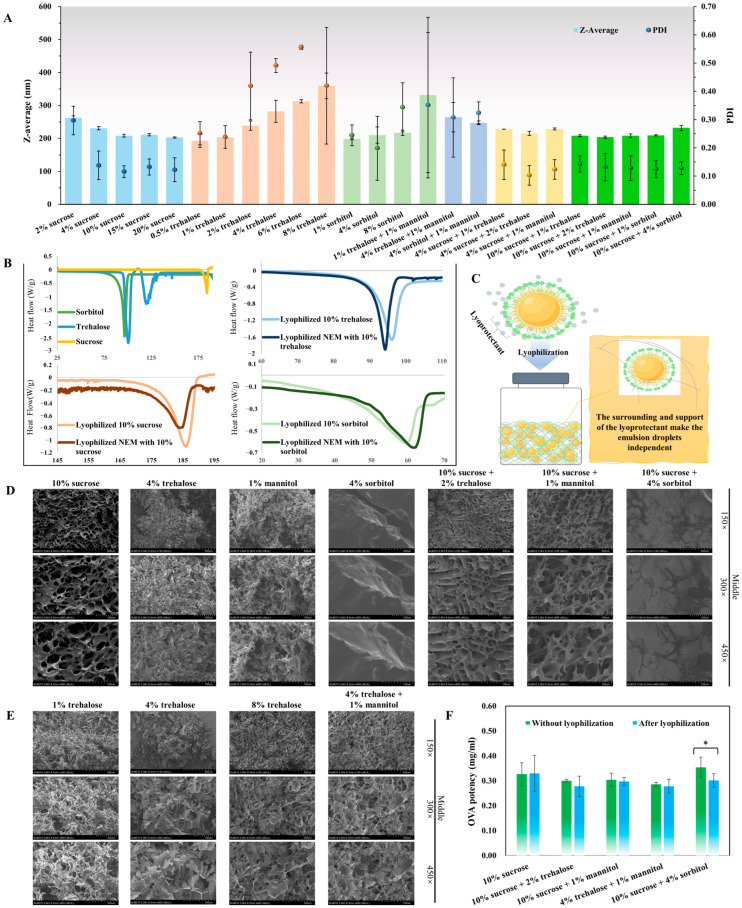
Comparison of the characteristics of reconstituted lyophilized powder emulsions with various lyoprotectants added. (**A**) Z-average particle size and PDI, (**B**) Heat flow profiles of lyophilized lyoprotectant cakes with and without NEM, (**C**) Presence of lyoprotectant creates closed holes to prevent emulsion fusion. SEM images of lyophilized NEMs (**D**) with 10% sucrose, 4% trehalose, 1% mannitol, 4% sorbitol, 10% sucrose + 4% trehalose, 10% sucrose + 1% mannitol, 10% sucrose + 4% sorbitol, 4% trehalose + 1% mannitol, and (**E**) with 1% trehalose, 4% trehalose, 8% trehalose in the middle section of cakes at 150×, 300×, 400× magnifications. (**F**) The potency results of NEMs containing OVA without lyophilization and after lyophilization. * *p* < 0.1.

Most of the samples lyophilized with excipients could form a nice cake shape, thus the PDI after reconstitution is the most important indicator for judging the quality of lyophilized samples. From the results (Figure 7A), the PDI of the reconstituted emulsions performed well with the PDI less than 0.3 when sucrose was used as the single excipient. When the sucrose concentration was greater than or equal to 10%, the particle size was significantly reduced to 210 nm with a PDI less than 0.15. When trehalose was used as a single excipient, the particle size increased continuously to greater than 300 nm as the concentration of trehalose increased, and its PDI was greater than 0.4, although trehalose as the lyoprotectant was able to form a product with a cake morphology. When sorbitol was used as the single excipient at concentrations between 1% to 8%, none of the lyophilized samples had a cake shape, even if the particle size after reconstitution was less than 220 nm.

For samples with two lyoprotectant components, those protected by 4% sucrose combined with 1% mannitol showed results similar to 4% sucrose alone. Both Z-average particle size and PDI of the product were optimized with the combination of 1% mannitol and trehalose. Lyophilized samples of NEM protected by 4% sorbitol and 1% mannitol also failed to form a cake shape. Higher mannitol concentrations are necessary for cake formation. When 10% sucrose was mixed with other low-concentration lyoprotectants, the lyophilized samples had better homogeneity after reconstitution with a smaller size distribution. It could be found that the addition of other excipients has less effect on the lyophilization of oil-in-water emulsions when the high-concentration sucrose is served as the basic lyoprotectant.

DSC was conducted to measure heat flow differentials between samples and reference materials under identical thermal conditions. Melting points were selected as the key parameter for lyoprotectant characterization due to their precision in detecting structural changes. The melting point changes shown in Figure 7B for lyoprotectant cakes with and without NEM were compared to evaluate intermolecular interactions potentially responsible for the observed Z-average particle size and PDI results of the reconstituted aqueous emulsions in Figure 7A.

Without lyophilization, melting points were measured at 184.3 °C for sucrose, 100.5 °C for trehalose (anhydrous form), and 96.6 °C for sorbitol. After lyophilization, values shifted to 185.97 °C (sucrose, ΔT = +1.67 °C), 96.5 °C (trehalose, ΔT = −4.0 °C), and 59.8 °C (sorbitol, ΔT = −36.8 °C). Except for sucrose, the melting points of trehalose and sorbitol decreased. This may occur because excipient powders exist in a highly crystalline state with tight molecular packing, requiring higher energy to disrupt the lattice. After lyophilization, ice sublimation may partially destroy the original crystal structure, forming an amorphous state. Amorphous materials exhibit loose molecular arrangement and weakened intermolecular forces, making them more susceptible to thermal disruption and thus lowering the melting point.

For lyophilized NEM systems, melting points remained statistically invariant (± 3 °C): 184.1 °C (NEM with sucrose), 94.3 °C (NEM with trehalose), and 62.0 °C (NEM with sorbitol). This indicates that despite NEM containing high concentrations of components (e.g., oil phase, surfactants), no significant interaction occurs between lyoprotectants and NEM during the lyophilization or reconstitution process. Therefore, differences in the physical structure of lyophilized cakes may need to be explored to understand how distinct lyoprotectants affect the maintenance of NEM’s particle size/PDI after lyophilization. SME images (Figure 7D) reflected the pore shape and structure of lyophilized emulsion with different lyoprotectants. The structure of the lyophilized emulsion with the addition of sucrose resembled a cork-like and closed circle, it had larger pore sizes, and a more homogenous pore distribution. Lyophilized emulsion with trehalose or mannitol only formed a few unclosed, continuous holes, most of which existed in overlapping, unsupported layered structures. When trehalose or mannitol was combined with sucrose, the boundaries of these holes became clearer and the size was more uniform. However, not all sucrose combinations would form the closed circle structure: cake still did not form when combined with sorbitol. Sorbitol has a lower collapse temperature (Tc), around −40 °C compared to sucrose and trehalose, which explains why the cake morphology could not form in lyophilization process, as the primary drying temperature was −25 °C. Hector and Deepark [45] also found collapsed and shrunken samples after lyophilization with the addition of a single sorbitol excipient. It is worth noting that the lyophilized emulsion had few supporting and closed holes when the trehalose density increased (Figure 7E). The trehalose tends to bind water during the freezing phase to form trehalose dihydrate, which limits its protective effect, particularly during ice formation [27]. This possibly explains the differences seen in the structures where hole edges became more vague as density increased.

Based on DSC and SEM results, it can be deduced that physical support from non-interactive forces plays a significant role in maintaining the spatial morphology and size of NEM during lyophilization. Uniform and appropriately sized pores can provide effective spatial isolation of individual droplets: pore walls and pore throats act as geometric barriers that limit droplet migration, thereby reducing droplet contact frequency and the probability of coalescence during reconstitution. In particular, the continuous and uniform porous structure formed in sucrose-containing lyophilized cake can provide instantaneous dispersion and stabilization of droplets during reconstitution. However, lyoprotectants such as the sorbitol failed to form an effective porous structure capable of supporting and isolating the emulsion droplets, rendering the emulsion more susceptible to phase separation, phase inversion, or phase fusion (Figure 7C). These hypothesis are collectively consistent with prior particle size and PDI performance.

In order to validate the hypothesis, biological studies were conducted using ovalbumin (OVA) as a model antigen. NEMs containing OVA and different lyoprotectants listed in Table 2 were lyophilized, followed by immediate in vitro potency assessment of both lyophilized and unlyophilized antigen solutions (control). The potency results (Figure 7F) revealed antigen activity in formulations (10% sucroce, 10% sucroce + 2% trehalose, 10% sucrose + 1% mannitol and 4% trehalose + 1% mannitol) relative to unlyophilized controls, whereas 10% sucroce + 4% sorbitol systems exhibited reduced potency (*p* < 0.1). This discrepancy aligns with SEM and Z-average particle size/PDI results, and is primarily driven by phase inversion/fusion events during lyophilization and reconstitution. These events promote either antigen entrapment within the oil core of NEMs or adsorption onto surfactant interfaces, thereby masking conformational epitopes critical for antibody binding.

### 3.4. Investigation of Freeze-Drying Process Parameters

Taguchi DOE methodology is a powerful tool for optimizing the process parameters in various biological experiments; it requires experienced personnel to select the control factors to be optimized and determine the appropriate levels for each selected factor [45]. The workflow of Taguchi DoE methodology consists of four sequential steps: selecting control factors and determining the appropriate levels for each factor based on experience, designing the experiment using the Taguchi technique, performing the designed experiments, and analyzing the experimental data using Minitab 21 software. Response data were read as a signal-to-noise (S/N) ratio. Then the maximum values of the S/N ratios were used for selecting the optimum levels for each factor.

**Figure 8:**
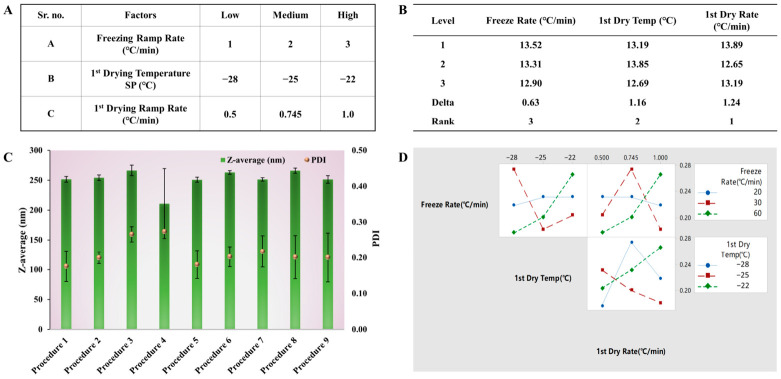
Process optimizing design and results with Taguchi. (**A**) Table of factors selected for optimizing PDI synthesis along with their respective levels, (**B**) Response table based on S/N ratio, (**C**) Experimental diagram showing the procedures used and the corresponding Z-average size and PDI, and (**D**) Interaction plot among factors.

Freezing rate is a critical parameter that needs to be optimized because different rates lead to the formation of various types of ice crystals. Rapid freezing produces small and numerous ice crystals, while slow freezing results in larger and fewer crystals. Specific surface area of the crystal will impact drying efficiency in the following steps. The heat required during primary drying is mainly supplied by the shelf and the shelf temperature is a critical parameter affecting the drying process. A suitable shelf temperature provides sufficient heat for sublimation, and ensures product quality. The heating rate and vacuum pressure during primary drying process are also factors that need to be considered, as suitable parameters can avoid damage to the product structure [46]. Considering the tested Tc value of NEM with 10% sucrose + 1% mannitol and the machine capability, three different factors each at three different levels were designed for optimizing the PDI representing the NEM’s homogeneity (Figure 8A), i.e., freezing ramp rate (3, 2, 1 °C/min), first drying temperature (−22, −25, −28 °C), and first drying ramp rate (1, 0.745, 0.5 °C/min). The layout of the L_9_ orthogonal array (OA) used in the present study is shown in Figure 8. Figure 8B represented the response table for S/N (signal to noise) ratio, where smaller values are preferred for the PDI. The interaction effect plot shown in Figure 8D represented the mean response at all possible combinations of any two factors. It was seen that there was a clear interaction between each of the two factors. In addition, based on the last two rows, delta values represented the impact ranking of the factor on the PDI outcome. In the present study, a larger S/N ratio was chosen as the criterion, as the goal was to maximize the response. The order in which each individual factor affects the PDI can be ranked as: first drying ramp rate > first drying temperature > freezing ramp rate. Eventually, first drying ramp rate at level 1, first drying temperature at level 2, and freezing ramp rate at level 1 were considered the best parameters for minimizing the PDI and thus optimizing the homogeneity of lyophilized NEM.

### 3.5. Comparative Stability Study of Freeze-Dried NEMs via IJM and HSHM

In order to further examine the feasibility and stability of lyophilized NEM, NEMs prepared by IJM and HSHM were investigated with the lyoprotectant of 10% sucrose and 10% sucrose + 1% mannitol, with the process parameters selected from the lyophilization process optimization.

**Figure 9:**
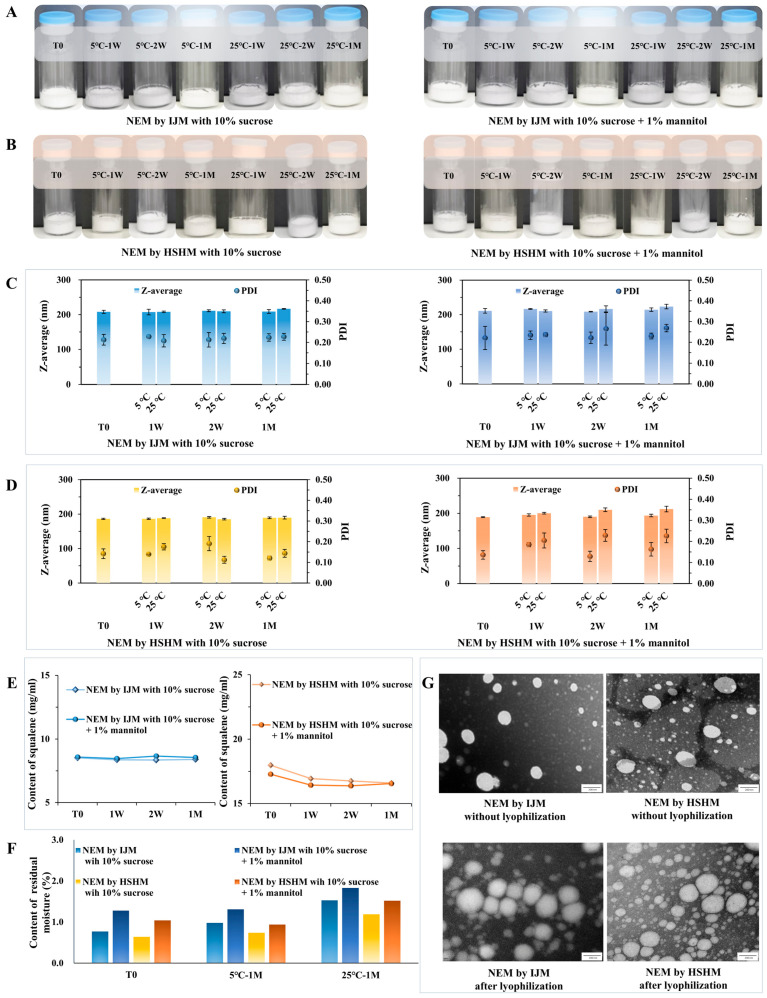
Stability results of lyophilized NEMs with the lyoprotectant of 10% sucrose and 10% sucrose + 1% mannitol. Cake morphology of NEMs by (**A**) IJM and (**B**) HSHM, Z-average size and PDI of NEMs by (**C**) IJM and (**D**) HSHM, (**E**) content of squalene and (**F**) content of residual moisture by IJM and HSHM, (**G**) TEM images of NEMs by IJM and HSHM without lyophilization and after lyophilization.

From the results shown in Figure 9A,B, both lyophilized NEMs prepared by IJM and HSHM presented intact cake morphology after lyophilization, without cracks, shrinkage, or collapse. All cakes maintained a stable shape for 1 week, 2 weeks, and 1 month at 5 °C and 25 °C. Upon reconstitution, all samples dissolved completely within 5 s and the rehydrated aqueous emulsion appeared as homogeneous milky white liquid. Moisture content in lyophilized cake is a critical parameter to evaluate the stability of lyophilized products: high residual moisture content can destabilize nanoparticles upon storage by inducing crystallization of cryoprotectants. It is generally considered that the stability of the cake improves when the residual moisture in the lyophilized cake is less than 3%. It could be found that all moisture results of the lyophilized NEM cakes were less than 3% even after 1 month of storage at 5 °C and 25 °C (Figure 9F). Moreover, when stored at different conditions for 1 week, 2 weeks, and 1 month at 5 °C and 25 °C, the Z-average size and PDI of lyophilized NEM cakes remained consistent after reconstitution (Figure 9C,D). The trends of squalene content of lyophilized NEMs prepared by IJM and HSHM (Figure 9E) after reconstitution showed the stable status under different conditions. Meanwhile, the TEM images of NEMs by IJM and HSHM without and after lyophilization revealed that both sample groups exhibited near-spherical and regular morphology with well-defined particle boundaries (Figure 9G). Image-based particle-size distributions showed no marked differences between the two groups, which was corroborated by DLS measurements, indicating that the NEMs produced by the two processes are comparable. The above stability test results fully demonstrate the feasibility of the lyophilized NEMs preparation process. Notably, the lyophilized NEMs prepared by both the IJM and HSHM methods exhibit excellent comparability across all parameters.

**Figure 10:**
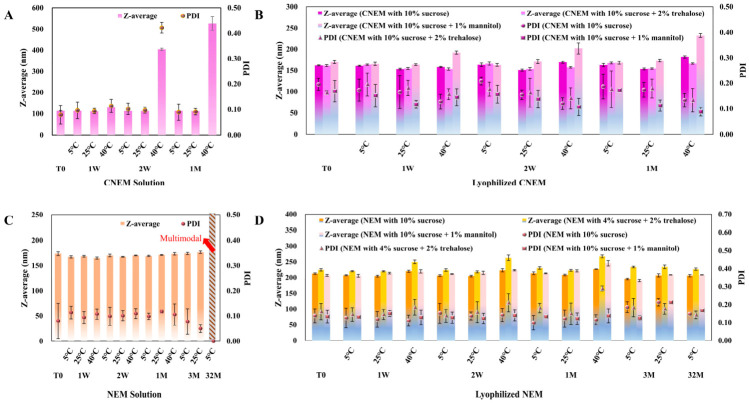
Stability results of CNEMs and NEMs with various lyoprotectants. CNEMs lyophilized with 10% sucrose, 10% sucrose + 2% trehalose, or 10% sucrose + 1% mannitol; NEMs lyophilized with 10% sucrose, 4% sucrose + 2% trehalose, or 10% sucrose + 1% mannitol. Comparison of CNEMs (**A**) without lyophilization and (**B**) after lyophilization, and NEMs (**C**) without lyophilization and (**D**) after lyophilization.

Finally, for validating the stability of lyophilized NEMs with the preferred and lyoprotectants and process, both of the NEMs and CNEMs were prepared with three sets of lyoprotectants listed in Table 2, followed by freeze-drying under optimized process parameters as detailed in Table 1. This study employed particle size and polydispersity index as primary physicochemical indicators to assess the impact of the lyophilization process on the colloidal stability and structural integrity of the NEM systems. From the results shown in Figure 10B, the lyophilized CNEMs formulated with optimized lyoprotectants remained relatively stable without significant upward trends in particle size after storage for 1 month at 5 °C, 25 °C, and even at 40 °C. The PDI values consistently remained below 0.3, indicating a narrow and uniform particle size distribution. In contrast, the CNEM solution without freeze-drying (Figure 10A) demonstrated a marked increase in particle size to approximately 400 nm, accompanied by a PDI exceeding 0.4 after 2 weeks at 40 °C under the accelerated conditions. Similarly, Figure 10D shows that lyophilized NEMs with optimized lyoprotectants maintained its particle size; even extended storage at 5 °C for 32 months did not result in a measurable change compared to the initial value. By contrast, non-lyophilized NEM stored at 5 °C for 32 months (Figure 10C) exhibited a multimodal particle size distribution, which precluded determination of a representative or accurately quantifiable particle size. A multimodal particle-size distribution indicated the presence of two or more distinct size populations, which often reflected instability mechanisms in the emulsion, including coalescence and Ostwald ripening (smaller particles dissolve and redeposit onto larger particles within a dispersed system, leading to the growth of larger particles at the expense of smaller ones) [47]. Therefore, all the results proved our previous hypothesis that applying the lyophilization technique could enhance the stability and shelf-life of the NEM system.

## 4. Conclusions

Through systematic optimization of the flow rate ratio, total flow rate, preparation temperature, and shearing mode, NEMs produced by IJM exhibited physical properties observed in the stability study that were comparable to those produced by HSHM. These findings indicate that IJM is a promising technique for NEM production. Future studies conducting comparative experiments on parallel processing—specifically comparing single-unit operation with multi-unit parallel operation modes—could enable its widespread application in production scale-up [48].

The optimized lyophilization technique makes the freeze-dried emulsion possible by forming closed holes to prevent the easy-to-flow monolayer from fusing. With the lyoprotectants screen, suitable types and combinations enabled consistence of lyophilized NEM exhibiting monodispersity (PDI < 0.2) and tight size distribution (200–300 nm), which governed its adjuvant efficacy. The study elucidated the underlying mechanisms for lyophilized NEMs with different lyoprotectans through analysis of intermolecular interactions (DSC) and morphological architecture (SEM) within lyophilized cakes, while employing model antigens for hypothesis validation. The mutually corroborative experimental results—exhibiting consistent trends—substantiate particle size/PDI as a primary evaluation metric for all the studies. Morever, bioactivity comparisons of without and after lyophilization confirm the feasibility of co-lyophilizing NEM-antigen systems for future commercialization.

In addition, lyophilization process parameters were optimized using a Taguchi approach, focusing on the freezing ramp rate, first drying temperature, and first drying ramp rate. The factors ranked by their impact on NEM homogeneity were: first drying ramp rate > first drying temperature > freezing ramp rate. With these optimized lyophilization parameters, lyophilized powders of NEMs produced by IJM and HSHM retained their critical physical and chemical attributes.

Analysis of stability-study data for liquid and lyophilized CNEMs and NEMs revealed that lyophilized samples maintained stable mean particle size and PDI under both accelerated and long-term storage conditions compared to their liquid formulations. It is well-established that low moisture content restricts molecular diffusion, thereby delaying various forms of chemical degradation and physical changes. Optimized lyoprotectants and lyophilization parameters formed a protective layer at interfaces during freeze-drying, reducing interfacial stress and particle–particle contacts of CNEMs and NEMs. They achieved low residual moisture and homogeneous, reconstitutable cakes, which inhibit coalescence, Ostwald ripening, and aggregation—the principal pathways leading to multimodal size distributions.

In conclusion, this study demonstrates the feasibility of using IJM as an alternative to HSHM for producing NEM, achieving comparable product quality while offering potential advantages such as lower contamination risk, smoother scale-up to continuous processing, and improved reproducibility. Converting the NEMs into lyophilized powders with optimized lyoprotectants and parameters significantly enhanced their stability and storage performance, thereby facilitating industrial application and supply-chain robustness. Moreover, the formulation and process insights gained here enrich the development knowledge base for single-dose, adjuvanted, lyophilized vaccines, directly supporting their translation into commercially viable products.

## Figures and Tables

**Table 1:** The lyophilization parameter used in different studies.

Process		Preliminary Study		Procedure Parameter Study									Lyoprotectant Agents Study	Stability Study
				Procedure 1	Procedure 2	Procedure 3	Procedure 4	Procedure 5	Procedure 6	Procedure 7	Procedure 8	Procedure 9		
Loading	Temperature/°C	22	22	22	22	22	22	22	22	22	22	22	22	22
Freezing	Temperature/°C	−60	−60	−60	−60	−60	−60	−60	−60	−60	−60	−60	−60	−60
	Ramp Time/°C/min	2	2	1	1	1	2	2	2	3	2	3	2	2
	Hold Time/h	6	6	6	6	6	6	6	6	6	6	6	6	6
Primary drying	Temperature/°C	4	−25	−28	−25	−22	−28	−25	−22	−28	−25	−22	−25	−25
	Chamber Vacuum/uBar	66	66	66	66	66	66	66	66	66	66	66	66	66
	Ramp Time/°C/min	0.745	0.745	0.50	0.745	1.00	0.50	0.745	1.00	0.50	0.745	1.00	0.5	0.5
	Hold Time/h	20	20	20	20	20	20	20	20	20	20	20	20	20
Secondary drying	Temperature/°C	20	20	20	20	20	20	20	20	20	20	20	20	20
	Chamber Vacuum/uBar	0.05	0.05	0.05	0.05	0.05	0.05	0.05	0.05	0.05	0.05	0.05	0.05	0.05
	Ramp Time/°C/min	0.75	0.75	0.75	0.75	0.75	0.75	0.75	0.75	0.75	0.75	0.75	0.75	0.75
	Hold Time/h	5	5	5	5	5	5	5	5	5	5	5	5	5

**Table 2:** The composition of lyoprotectant agents in the lyoprotectant agent study and stability study.

System	Lyoprotectant	Concentration *w*/*v*	System	Lyoprotectant	Concentration *w*/*v*
Lyoprotectant agent study					
NEM	Sucrose	2%	NEM	Trehalose + Mannitol	1% + 1%
	Sucrose	4%		Sorbitol + Mannitol	4% + 1%
	Sucrose	15%		Sucrose + Trehalose	4% + 1%
	Sucrose	20		Sucrose + Trehalose	4% + 2%
	Trehalose	0.5%		Sucrose + Mannitol	4% + 1%
	Trehalose	1%		Sucrose + Trehalose	10% + 1%
	Trehalose	2%		Sucrose + Sorbitol	10% + 1%
	Trehalose	4%	NEM NEM + OVA		
	Trehalose	6%		Sucrose	10%
	Trehalose	8%		Sucrose + Trehalose	10% + 2%
	Sorbitol	1%		Sucrose + Mannitol	10% + 1%
	Sorbitol	4%		Trehalose + Mannitol	4% + 1%
	Sorbitol	8%		Sucrose + Sorbitol	10% + 4%
Stability study					
NEM	Sucrose	10%	CNEM	Sucrose	10%
	Sucrose + Mannitol	10% + 1%		Sucrose + Mannitol	10% + 1%
	Sucrose + Trehalose	10% + 2%		Sucrose + Trehalose	4% + 2%

## Data Availability

The original contributions presented in this study are included in the article. Further inquiries can be directed to the corresponding author.

## Abbreviations

The following abbreviations are used in this manuscript:

NEM	Nanoemulsion
IJM	Impingement jet mixing
DOE	Design of Experiment
PDI	Polydispersity index
W/O	Water-in-oil emulsion
O/W	Oil-in-water emulsion
O/W/O	Oil-in-water-in-oil emulsion
W/O/W	Water-in-oil-in-water emulsion
DAMP	Damage-associated molecular patterns
CNEM	Cationic nanoemulsion
Tween 80	Polyoxyethylene sorbitan monooleate
Span 85	Sorbitan trioleate
saRNA-CNEM	Self-amplifying RNA delivered by cationic nanoemulsion
HSHM	High-shear homogenization and then microfluidization
GMP	Good manufacturing practice
mRNA-LNPs	mRNA encapsulated within lipid nanoparticles
Span 80	Sorbitan monooleate
TFFD	Thin-film freeze-drying
TEM	Transmission electron microscopy
SEM	Scanning electron microscopy
DOTAP	1,2-dioleoyl-3-trimethylammonium-propane
ELSD	Evaporative light scattering detector
DSC	Differential scanning calorimetry
OVA	Ovalbumin
HRP	Horseradish peroxidase
TMB	3,3′,5,5′-tetramethylbenzidine
